# Supplementary material for: Pneumocystis Exacerbates Inflammation and Mucus Hypersecretion in a Murine, Elastase-Induced-COPD Model
Source: J Fungi (Basel). 2023 Apr 7;9(4):452. doi: 10.3390/jof9040452 (PMC10142929; doi:10.3390/jof9040452)
Supplement: Supplementary file 1 [file jof-09-00452-s001.zip › jof-2296630-supplementary.pdf]

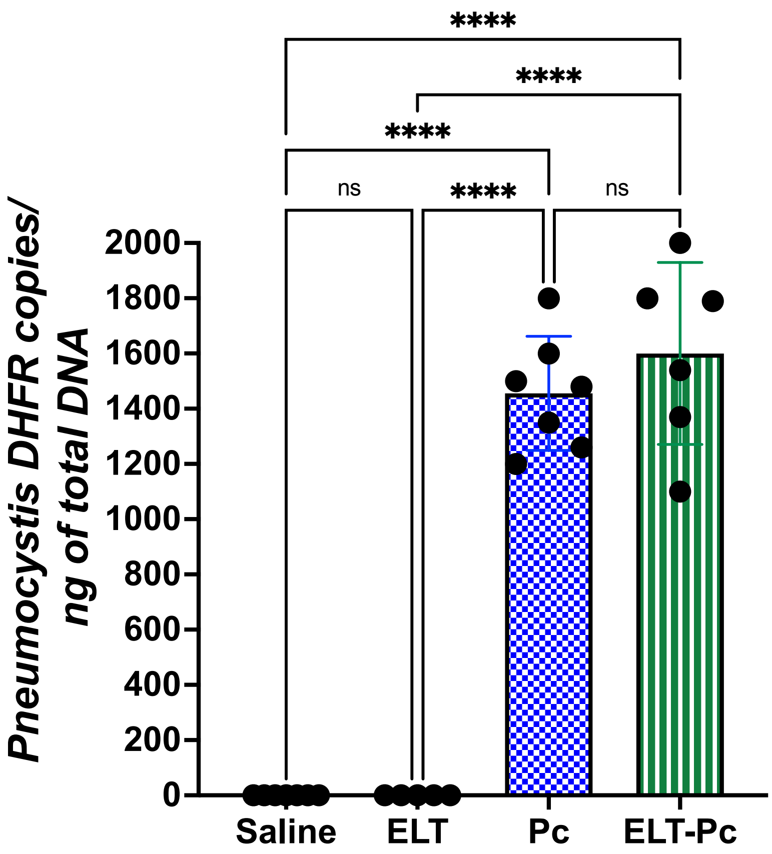

Figure S1. Quantification of the burden of *Pneumocystis*. *dhfr* gene levels were evaluated by qPCR. Data were determined using interpolation of each Ct in a calibration curve. Data were expressed as mean±SD and were adjusted to 100 ng of total genomic DNA for each sample. All data was analyzed by ANOVA. ns = non-significant; **** = p<0.0001.
